# Yap1 plays a protective role in suppressing free fatty acid-induced apoptosis and promoting beta-cell survival

**DOI:** 10.1007/s13238-016-0258-5

**Published:** 2016-03-22

**Authors:** Yaoting Deng, Yurika Matsui, Wenfei Pan, Qiu Li, Zhi-Chun Lai

**Affiliations:** Department of Biochemistry and Molecular Biology, Pennsylvania State University, University Park, PA 16802 USA; Intercollege Graduate Degree Program in Molecular, Cellular and Integrative Biosciences, Pennsylvania State University, University Park, PA 16802 USA; Shandong Provincial Hospital Affiliated to Shandong University, Jinan, 250021 China; Department of Biology, Pennsylvania State University, University Park, PA 16802 USA

**Keywords:** β-cell, CTGF, F-actin, free fatty acid, Hippo signaling, Yap1

## Abstract

Mammalian pancreatic β-cells play a pivotal role in development and glucose homeostasis through the production and secretion of insulin. Functional failure or decrease in β-cell number leads to type 2 diabetes (T2D). Despite the physiological importance of β-cells, the viability of β-cells is often challenged mainly due to its poor ability to adapt to their changing microenvironment. One of the factors that negatively affect β-cell viability is high concentration of free fatty acids (FFAs) such as palmitate. In this work, we demonstrated that Yes-associated protein (Yap1) is activated when β-cells are treated with palmitate. Our loss- and gain-of-function analyses using rodent insulinoma cell lines revealed that Yap1 suppresses palmitate-induced apoptosis in β-cells without regulating their proliferation. We also found that upon palmitate treatment, re-arrangement of F-actin mediates Yap1 activation. Palmitate treatment increases expression of one of the Yap1 target genes, connective tissue growth factor (CTGF). Our gain-of-function analysis with CTGF suggests CTGF may be the downstream factor of Yap1 in the protective mechanism against FFA-induced apoptosis.

**Electronic supplementary material:**

The online version of this article (doi:10.1007/s13238-016-0258-5) contains supplementary material, which is available to authorized users.

## INTRODUCTION

 Pancreatic β-cells synthesize and secrete insulin for glucose homeostasis, and decrease in the number of functional β-cells results in type 1 and type 2 diabetes. Chronically increased level of free fatty acids (FFAs) in plasma is linked to type 2 diabetes (T2D) by causing both insulin resistance and β-cell failure (Boden and Shulman, 2002; Boden, 2008). In the pathogenesis of T2D, peripheral organs first become less sensitive to insulin. In order to compensate for this resistance, β-cells increase the synthesis and secretion of insulin. This, however, leads to β-cell dysfunction and/or death and the insulin level become inadequate for a glucose challenge (Kusminski et al., 2009; Haber et al., 2003; Haber et al., 2006; Marchetti et al., 2004; Vetere et al., 2014). Improving the treatment of diabetes has been challenging due to the limited proliferation of β-cells and their poor ability of adapting to cytotoxic environments. However, several therapeutic approaches for diabetes have been taken to increase or maintain the number of β-cells by inducing proliferation of β-cells, protecting β-cells from cell death and re-programming other cell types to β-cells (Vetere et al., 2014).

One of the consequences of chronic exposure to the high level of FFAs is β-cell apoptosis. Among FFAs, saturated FFAs such as palmitate, one of the most abundant FFAs in plasma, shows higher toxicity on β-cells than unsaturated FFAs such as oleate (Eitel et al., 2002; Karaskov et al., 2006; Maedler et al., 2001). FFAs induce apoptosis in β-cells through multiple complex mechanisms. Studies have shown that FFAs induce oxidative stress such as nitric oxide production, leading to mitochondrial DNA (mtDNA) damage and apoptosis. Ceramide, a fatty acid-containing secondary messenger, has also been reported to mediate FFA-induced apoptosis in diabetic rats and primary islets treated with FFAs (Shimabukuro et al., 1998). Additionally, FFAs reduce Ca^2+^ uptake by endoplasmic reticulum (ER) and causes ER stress followed by the activation of c-Jun N-terminal kinase (JNK) (Cunha et al., 2008; Eizirik et al., 2008; Karaskov et al., 2006). FFA receptors such as G protein-coupled receptor 40 (GPR40), which play an essential role in FFA-induced insulin secretion under physiological condition, could also mediate FFA-induced apoptosis (Natalicchio et al., 2013). Furthermore, signaling pathways that may transduce apoptotic signals from FFAs have been investigated. JNK and p38 Mitogen-activated protein kinase (MAPK) pathways become activated upon FFA treatment and the activation is at least partially controlled by GPR40, Mitogen-activated protein kinase kinases 4/7 (MKK4/7) and/or protein kinase A (PKA) (Natalicchio et al., 2013).

Yes-associated protein (Yap1) is a transcriptional co-activator which regulates cell proliferation, differentiation and apoptosis. Yap1 protein has a proline-rich region at its N-terminus, a TEAD-binding region, one or two WW domains, an SH3-binding motif, a coiled-coil domain and a PDZ interaction motif at its C-terminus (Zhao et al., 2010). In order to execute its function as a transcriptional co-activator, Yap1 binds to DNA-binding transcription factors, and the interaction with different DNA-binding factors results in different cellular responses. Identification of genes that Yap1 regulates has been growing in number; for instance, baculoviral IAP repeat containing 2/5 (Birc 2/5) inhibit apoptosis, and connective tissue growth factor (CTGF) and Axl receptor tyrosine kinase (Axl) promote proliferation (Zhao et al., 2008; Zhao et al., 2010). In addition to Yap1’s function as an oncogene, it can induce apoptosis by associating with p73, a functional homolog of p53, and up-regulate the expression of pro-apoptotic genes, such as Bcl2-associated X protein (Bax) and promyelocytic leukemia (PML) (Basu et al., 2003; Lapi et al., 2008; Zhang et al., 2011).

In the pancreas, Yap1 is one of the factors that determine the organ size and the composition of different cell types. At the early stage of pancreatic development, the pancreatic epithelium is made up of progenitor cells that later differentiate into endocrine, acinar and ductal cells. Yap1 is highly expressed throughout the pancreas during this stage (George et al., 2012). Later in the pancreatic development, the progenitor cells actively proliferate and differentiate into the three different cell types. Yap1 is now expressed in the more restricted area of pancreas where ductal- or acinar-fated cells are present (George et al., 2012). Several lines of evidence have shown that Yap1 is an essential factor regulating proliferation and differentiation during this later stage of pancreatic development (Camargo et al., 2007; Gao et al., 2013). Conditional overexpression of Yap1 in mice at this stage interfered with the differentiation program, causing the increase in the ductal cell population, while reducing the populations of acinar and endocrine cells (Gao et al., 2013). Moreover, chimeric mice with the doxycycline-induced overexpression of Yap1 increased the size of the pancreas with more acinar cells (Camargo et al., 2007). In the adult pancreas, Yap1 expression is further limited to ductal and terminal-duct centroacinar cells with weak expression in acinar cells and undetectable expression in endocrine cells (George et al., 2012).

In this study, we explored the role of Yap1 in rodent insulinoma cell lines. Weak expression of Yap1 was detected in these insulinoma cell lines. Using loss- and gain-of-function analyses, we demonstrated that Yap1 is essential for the maintenance of β-cell viability both under FFA-treated and untreated conditions. Upon FFA treatment, Yap1 becomes activated and antagonizes FFA-induced apoptosis. This is partly done by increasing the expression of CTGF. Factors like CTGF may go on to play a protective role in promoting β-cell survival. Such knowledge could be potentially useful for coming up with therapeutic strategies for treating T2D.

## RESULTS

### Free fatty acid, palmitate, induces apoptosis of pancreatic β-cells through F-actin remodeling

**Figure 1:**
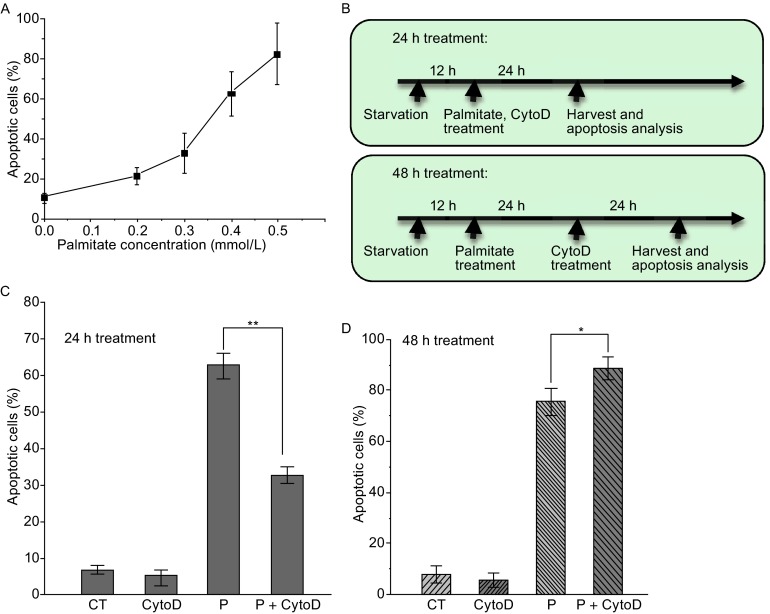
Palmitate induces β-cells apoptosis through F-actin remodeling. (A) Apoptosis of rat INS-1 832/13 cells was induced with palmitate at different concentrations for 24 h. Apoptotic cells were recognized as Annexin V-positive and PI-negative cells and quantified through flow cytometry. (B) Time lines along with specific events for the 24-h and 48-h palmitate treatment procedures. (C) CytoD reduced palmitate-induced apoptosis under the 24-h treatment as quantified with the Annexin V assay. (D) CytoD slightly enhanced palmitate-induced apoptosis under the 48-h treatment. In (C) and (D), final concentrations of palmitate (0.3 mmol/L) and CytoD (0.5 µmol/L) were used, and data show the mean ± SD of four independent experiments. CT: Control; CytoD: Cytochalasin D; P: Palmitate. Ethanol (solvent of palmitate) and DMSO (solvent of CytoD) were used in the control experiments. “*” and “**” indicate *P* < 0.05 and *P* < 0.01, respectively, by Student’s *t*-test

Previous studies have shown that saturated free fatty acids (FFAs) such as palmitate can induce mammalian pancreatic β-cells to undergo apoptosis (Kusminski et al., 2009). Using rat INS-1 832/13 cells, we found that palmitate caused cell apoptosis in a dosage-dependent manner (Fig. 1A). In this experiment, cells were starved for 12 h before palmitate treatment. After 24 h of palmitate treatment, cells were harvested and the population of cells undergoing apoptosis was quantified through the Annexin V-based assay (Fig. 1B). Annexin V binds to phosphatidylserine (PS) presented on the outer leaflet of the plasma membrane of apoptotic and dead cells, whereas propidium iodide (PI) is a nucleic acid dye which can only stain dead cells due to the disrupted membrane. Therefore, early apoptotic cells can be identified as Annexin V-positive and PI-negative cells. Oleate, an unsaturated FFA, exhibited a similar effect on the INS-1 832/13 cells (data not shown).

F-actin arrangement is critical for maintaining β-cell metabolic function (Kalwat and Thurmond, 2013). To explore how palmitate induces apoptosis, we tested the involvement of F-actin by disrupting F-actin polymerization with Cytochalasin D (CytoD) in INS-1 832/13 cells. We found that CytoD treatment reduced palmitate-induced apoptosis when CytoD was applied together with palmitate for 24 h (Fig. 1C). Another F-actin inhibitor, Latrunculin B (LatB), exhibited a similar effect (data not shown). This suggests that palmitate can function through actin cytoskeleton to transduce its apoptotic signal in β-cells. Interestingly, after a prolonged treatment (48 h) with CytoD subsequent to palmitate induction (Fig. 1B, the bottom panel), CytoD was no longer effective for this inhibition. Instead, it even enhanced the palmitate-induced apoptosis (Fig. 1, compare D with C). Analysis of activated Caspase 3 provided consistent results (Fig. S1). Thus, F-actin is critical for leading the cells to palmitate-induced apoptosis; however, once the apoptotic program is initiated, disruption of actin cytoskeleton does not arrest the apoptotic event.

### Yap1 is activated in palmitate-treated β-cells

**Figure 2:**
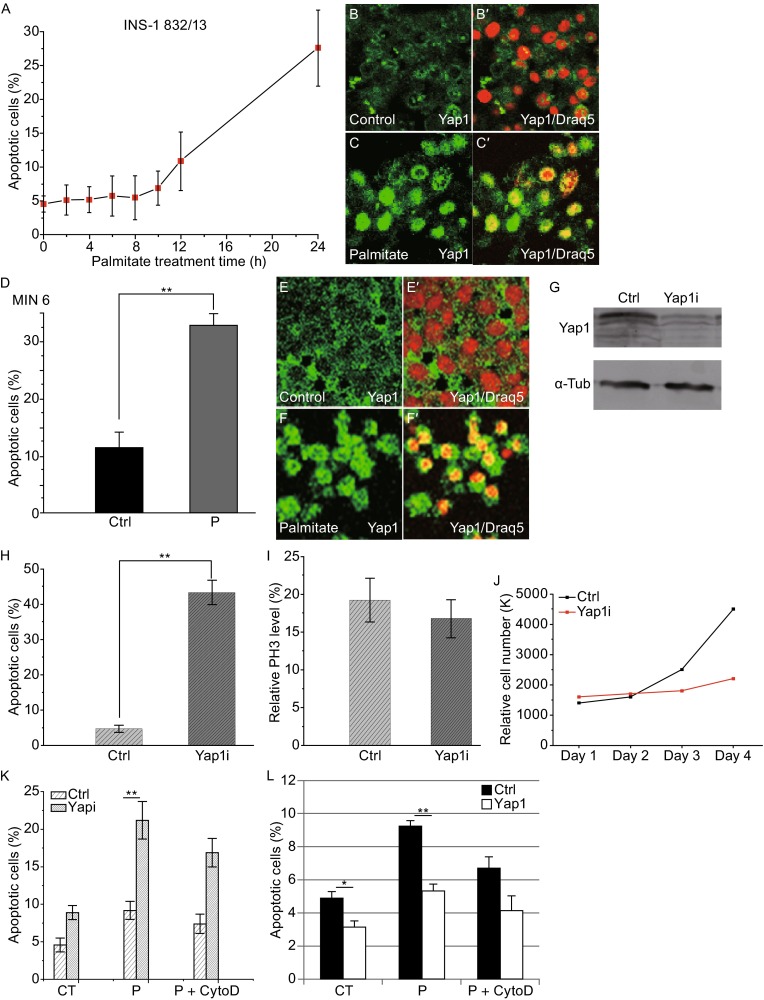
Yap1 is translocated to the nucleus upon palmitate treatment to promote β-cell survival. (A) For rat INS-1 832/13 cells, apoptosis began to increase after 10 h of palmitate (0.3 mmol/L) treatment. Apoptosis was analyzed at indicated time points via the Annexin V assay. Data show the mean of three independent experiments. (B-B′, C-C′) Immuno-staining of the β-cells showed that Yap1 translocated and accumulated in the nucleus in most cells after 24 h of palmitate treatment. Draq 5 staining was used to identify nuclei in (B′ and C′). Yap1 localization was observed every two hours and Yap1 began to respond to palmitate treatment after 12 h, later than the initiation of apoptosis. (D) For mouse MIN6 β-cells, apoptosis was observed after 48 h of treatment with 0.5 mmol/L palmitate. Apoptosis was measured with the Annexin V assay. Data show the mean ± SD of four independent experiments. (E-E′, F-F′) In MIN6 cells, Yap1 translocated to the nucleus in most cells after the 48-h palmitate treatment (F-F′), whereas Yap1 was more evenly distributed in untreated cells (E-E′). Draq 5 is a DNA dye. (G) Western blot analysis of Yap1 showed the Yap1 shRNA-mediated knockdown worked effectively to reduce Yap1 expression. (H) Yap1 knockdown promoted apoptosis. Cells were incubated with Yap1 shRNA-virus for 5 days in this experiment. (I) Yap1 knockdown did not significantly influence β-cell proliferation (*P* > 0.05). The population of PH3-positive cells was quantified by flow cytometry after staining with PH3 antibody. (J) Cell counting experiment showed that the increase of cell number over time was inhibited by the reduction of Yap1. Cell number was counted on a daily basis for four days. (K) Yap1 knockdown enhanced apoptosis after the 24-h palmitate treatment. Yap1 shRNA-virus was incubated with cells for 2 days in this experiment. (L) Yap1 overexpression decreased apoptosis with (*P* < 0.01) or without (*P* < 0.05) 24-h palmitate treatment. Lentivirus overexpressing human Yap1 was incubated for 3 days before 12-h serum starvation followed by the 24-h palmitate treatment. For (G–K), Yap1i: Yap1 shRNA; Ctrl: negative control. Data show the mean ± SD of four independent experiments. For (L), Yap1: human Yap1 overexpression; Ctrl: empty vector control. Solvents, ethanol and DMSO alone, were used in the control experiments (CT). CytoD: Cytochalasin D; P: Palmitate. “*” and “**” indicate *P* < 0.05 and *P* < 0.01, respectively, by Student’s *t*-test

 One of the genes that are involved in both F-actin remodeling and apoptosis in many cell types is Yap1. Yap1 can serve as an inducer or suppressor of apoptosis by regulating its target gene expression involved in apoptosis (Basu et al., 2003; Lapi et al., 2008; Zhao et al., 2008; Zhang et al., 2011). In addition, Yap1 and F-actin can regulate each other; accumulation of F-actin activates Yap1, while overexpression of constitutively active Yap1 increases F-actin level (Matsui and Lai, 2013; Moroishi et al., 2015). To investigate the function of Yap1 in mammalian β-cells under a stress condition, we induced apoptosis by palmitate in INS-1 832/13 cells and monitored the activity of Yap1 at different stages of apoptosis. Apoptosis began to increase after 10 h of palmitate treatment and after 24 h, apoptosis increased significantly (Fig. 2A). The activity of Yap1 was demonstrated by its subcellular localization. After 12 h of palmitate treatment, Yap1 translocated from cytoplasm to the nucleus. After 24 h, Yap1 was located in the nucleus in most β-cells (Fig. 2C-C′), whereas Yap1 was more localized in cytoplasm in the untreated cells (Fig. 2B-B′). Yap1 remained in the nucleus even after 48 h of palmitate treatment (data not shown). These results indicate that Yap1 is activated after palmitate-induced apoptosis. This increase in the Yap1 activity upon palmitate treatment was also found in its phosphorylation level. Western blot analysis demonstrated that the phosphorylation level of Yap1 at the conserved Ser127 site decreased after 24 h of palmitate treatment (Fig. S2A). Similar observations about the subcellular localization of Yap1 were made with mouse MIN6 β-cells (Fig. 2D-F′), indicating that it might be a general response for Yap1 to be activated after palmitate-induced apoptosis in mammalian β-cell lines.

### Yap1 promotes β-cell survival under palmitate-treated conditions

In most studies, Yap1 has been shown to promote cell proliferation and inhibit apoptosis by interacting with transcription factors such as TEAD1 (Pan, 2010; Staley and Irvine, 2012; Yu and Guan, 2013). However, in some cell types, Yap1 appears to promote apoptosis through association with a transcription factor, p73 (Basu et al., 2003; Lapi et al., 2008; Zhang et al., 2011). In order to decipher the role of Yap1 in β-cells, we carried out loss- and gain-of-function experiments of Yap1 in INS-1 832/13 cells. In the RNA interference approach, Yap1 knockdown enhanced apoptosis of INS-1 832/13 cells even without any treatment (Fig. 2H). This indicates the requirement of Yap1 in maintaining the viability of β-cells at normal condition. Using a phospho-Histone 3 (PH3)-specific antibody to recognize mitotic cells, we found that reduction of Yap1 had no significant effect on cell proliferation (Fig. 2I). Cell number counting showed that the rate of increase in cell number became significantly low when Yap1 level was reduced (Fig. 2J). Taken together, the increase in cell number was prevented in the absence of Yap1 mainly due to increased apoptosis. Importantly, when Yap1 was knocked down along with palmitate treatment, the apoptotic cell population increased synergistically (Fig. 2K). To further test if Yap1 is sufficient to regulate cell viability, we overexpressed human Yap1 in INS-1 832/13 cells. Yap1 overexpression decreased the population of apoptotic cells with or without palmitate treatment. We also observed that the increase in apoptosis upon palmitate treatment became less significant in cells with Yap1 overexpression (Fig. 2L). This suggests that Yap1 may provide a protective function against palmitate-induced apoptosis.

### Yap1 activity is regulated by F-actin dynamics upon palmitate treatment

**Figure 3:**
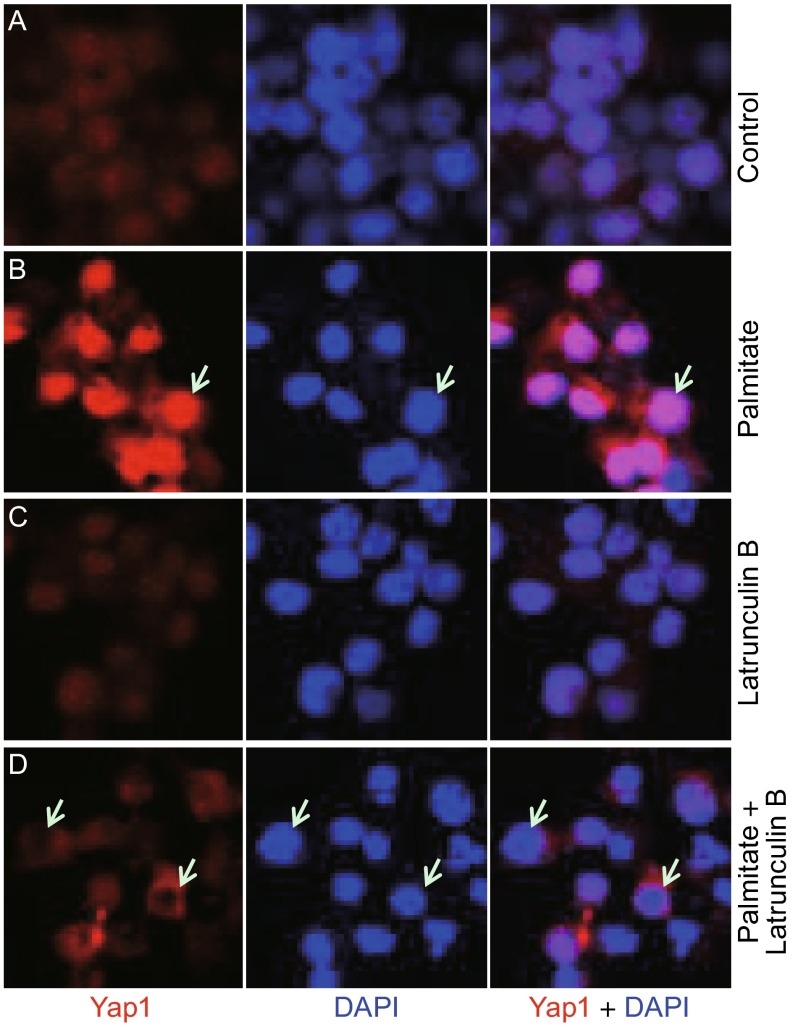
FFA-induced Yap1 nuclear localization is dependent on F-actin. Rat INS-1 832/13 cells were starved in the absence of serum for 12 h followed by the 24-h treatment with palmitate or/and Latrunculin B. (A) Control with solvents, DMSO and ethanol; (B) Palmitate (0.3 mmol/L); (C) Latrunculin B (1 µg/mL); (D) Palmitate (0.3 mmol/L) together with Latrunculin B (1 µg/mL). Yap1 was detected by an anti-Yap1 antibody through immunostaining. DAPI stains nucleic acids and served as a nuclear marker. White arrows show examples of nuclei that accumulate high levels of Yap1 in (B), or avoid Yap1 nuclear localization in (D)

To test whether Yap1 responds to FFA signaling through F-actin, F-actin polymerization was blocked by inhibitors such as CytoD or LatB. INS-1 832/13 cells were exposed to either palmitate alone or together with the F-actin inhibitors for 24 h following the 12-h serum starvation. The result showed that palmitate induced nuclear accumulation of Yap1 (Fig. 3, compare B with A), while LatB treatment alone did not affect the localization of Yap1 (Fig. 3C). However, when cells were treated with palmitate along with LatB, the translocation of Yap1 into the nucleus was partially blocked (Fig. 3, compare D with B). CytoD exhibited a similar effect (data not shown). Western blot analysis showed that CytoD inhibited dephosphorylation of Yap1 at Ser127 induced by palmitate treatment (Fig. S2A). These results indicate that F-actin is critically involved in Yap1 activation in responding to FFA treatment.

Recent studies both in mammalian and *Drosophila* systems showed that F-actin dynamics can be regulated by YAP1 (Yorkie in flies) (reviewed in Matsui and Lai, 2013; Moroishi et al., 2015). In order to test whether Yap1 regulates F-actin dynamics and provides feedback in β-cells, Yap 1 was knocked down by RNAi approach and the level of F-actin was quantified in INS-1 832/13 cells by flow cytometry. We found that the reduction of Yap1 did not have any effect on F-actin dynamics (Fig. S2B), and therefore, Yap1 does not appear to use a feedback mechanism to influence the dynamics of F-actin in β-cells.

### Expression of CTGF is activated by palmitate treatment in a Yap-dependent manner

**Figure 4:**
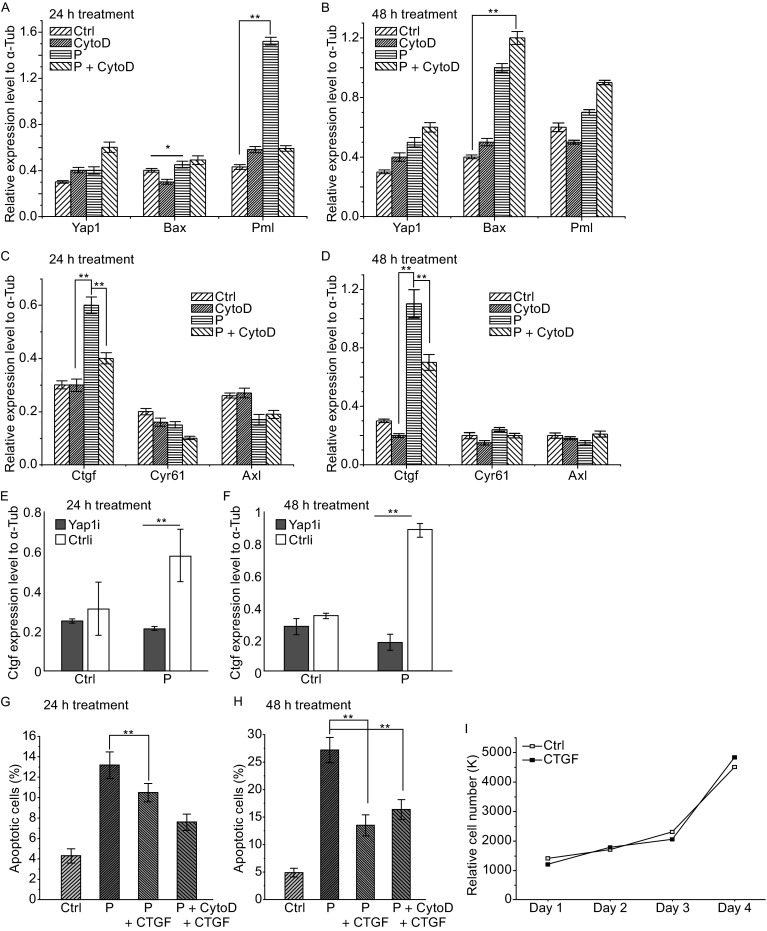
Analysis of Yap1 target gene expression and effect of CTGF on β-cell viability under palmitate treatment. Expression of several Yap1 target genes was measured by quantitative RT-PCR. Rat INS-1 832/13 β-cells were treated with palmitate and CytoD following either the 24-h or 48-h treatment procedure, as described in Fig. 1B. (A and B) Yap1/p73 target genes Bax and Pml showed no correlation with Yap1 activities under palmitate and CytoD treatment. (C and D) Yap1/Tead1 target gene CTGF showed a consistent expression level with Yap1 activity regulation by palmitate and CytoD treatment. (E and F) Yap1 knockdown repressed CTGF overexpression under palmitate treatment under both 24-h (E) and 48-h (F) conditions. Data show the mean ± SD of three independent experiments. For (A–F), solvent, ethanol, was used as control. CytoD: Cytochalasin D; P: palmitate; Yap1i: Yap1 shRNA; Ctrl: negative control. (G and H) Human CTGF inhibited palmitate-induced apoptosis under both the 24-h and 48-h treatments. Final concentrations of 1 µg/mL of CTGF, 0.3 mmol/L of palmitate and 0.5 µmol/L of CytoD were used. Under the 48-h treatment condition, CTGF inhibited apoptosis enhancement triggered by CytoD. Apoptosis was measured via the Annexin V assay. Solvents (ethanol, DMSO and NaOAC) were used in the control experiments. Data showed the mean ± SD of three independent experiments. (I) Cell counting experiment shows that CTGF (1 µg/mL) incubation has no obvious effect on the β-cell number. The number of cells was counted every day for four days after the treatment of exogenous CTGF. “*” and “**” indicate *P* < 0.05 and *P* < 0.01, respectively, by Student’s *t*-test

Yap1 functions as a transcription co-activator by interacting with DNA-binding proteins such as TEA-domain proteins (TEAD) to promote proliferation and inhibit apoptosis (Pan, 2010; Staley and Irvine, 2012; Yu and Guan, 2013). When associated with p73 transcription factor, Yap1 can promote apoptosis (Basu et al., 2003; Lapi et al., 2008; Zhang et al., 2011). We next investigated the expression of which genes are responsive to Yap1 in mammalian β-cells. INS-1 832/13 cells were treated with palmitate and CytoD separately or in combination. Expression levels of several Yap1/p73 and Yap1/TEAD1 target genes were monitored by quantitative reverse transcription-polymerase chain reaction (RT-PCR). It turned out that a p73 target gene, Bax, which is a pro-apoptosis gene, had no obvious change after 24 h of palmitate treatment; however, its expression was increased after 48 h. Bax expression was not influenced by CytoD (Fig. 4A and 4B). The expression of Pml, which is also an apoptosis-related gene, was up-regulated under 24-h palmitate treatment and this up-regulation was dependent on F-actin (Fig. 4A). However, Pml expression dropped after 48 h (Fig. 4B). Although expression levels of Bax and Pml were influenced by palmitate treatment, their expression patterns were not tightly correlated with Yap1 activity.

Expression of several TEAD1 target genes that include CTGF, Cyr61 and Axl was also examined. Cyr61 and Axl expression levels had no significant change after palmitate treatment at either 24-h or 48-h time points (Fig. 4C and 4D). However, the expression level of CTGF closely followed the activation pattern of Yap1 upon palmitate treatment. Under both 24-h and 48-h treatments, CTGF expression was up-regulated by palmitate, and this up-regulation was inhibited by CytoD in both cases (Fig. 4C and 4D). Therefore, the CTGF expression pattern is consistent with Yap1 activity, which is activated by palmitate, yet inhibited by CytoD. To investigate the importance of Yap1 for palmitate-induced CTGF up-regulation, a loss-of-function experiment was carried out through RNAi approach. When Yap1 level was reduced, palmitate-induced up-regulation of CTGF expression was blocked (Fig. 4E and 4F). Interestingly, without the palmitate treatment, Yap1 knockdown did not influence the basal expression level of CTGF (Fig. 4E and 4F). Taken together, these results support that CTGF is a downstream target of Yap1 in β-cells under palmitate treatment.

### CTGF inhibits FFA-induced apoptosis of β-cells

CTGF is highly expressed in mouse embryonic β-cells and functions to promote cell proliferation (Gunasekaran et al., 2012). In adult islets, CTGF expression level is decreased dramatically, and CTGF no longer promotes cell proliferation (Gunasekaran et al., 2012). However, high level of CTGF expression is still detected in adult ducts or the vasculature, suggesting that CTGF might still be involved in the maintenance of islet function in adult pancreas (Crawford et al., 2009). Because CTGF turned out to be the best candidate for a target gene of Yap1 in β-cells, we tested whether CTGF is able to enhance β-cell viability under palmitate treatment. INS-1 832/13 cells were treated with palmitate and CytoD by following the 24-h or 48-h treatment procedures (Fig. 1B), and human recombinant CTGF was added to the culture medium together with palmitate. β-Cells were harvested for apoptotic analysis by flow cytometry after the treatments. Both 24-h and 48-h palmitate treatments dramatically increased the apoptotic cell population. Human CTGF partially suppressed the palmitate-induced apoptosis (Fig. 4G and 4H). Under the 48-h treatment, we observed more potent anti-apoptotic effect of CTGF; CTGF inhibited apoptosis even in the presence of F-actin inhibitor, CytoD (compare Fig. 4H with Fig. 1D). This set of data suggests CTGF can enhance β-cell survival under palmitate treatment and act downstream of F-actin modulation.

Although endogenous expression of CTGF has been reported to be low in adult β-cells, whether β-cells are still responsive to exogenous CTGF remains unclear. To test whether CTGF exhibit any effect on β-cell growth, INS-1 832/13 cells were incubated with human CTGF and cell number was counted over a period of four days. Interestingly, CTGF incubation did not significantly influence the growth profile of β-cells (Fig. 4I). Thus, CTGF does not appear to dramatically affect survival and proliferation of β-cells in the absence of palmitate-induced apoptosis.

## DISCUSSION

Type 2 diabetes is strongly linked to obesity, which is associated with elevated levels of FFAs in blood. Chronically elevated FFA promotes insulin resistance and β-cell death (Morgan and Dhayal, 2009; Prentki and Madiraju, 2011). Therefore, a better understanding of how FFA signaling leads to β-cell dysfunction and death would contribute to the development of strategies for proper management and treatment of diabetes. In this study, we explored FFA-induced β-cell apoptosis and factors that can increase β-cell survival against this cytotoxic microenvironment using rodent insulinoma cell lines.

As Yap1 is critical in regulating cell proliferation and apoptosis in many mammalian cell types, we investigated whether the Yap1 activity is required in β-cells. Through immunofluorescence, we detected the expression of Yap1 protein in rodent insulinoma cell lines, such as INS-1 832/13 and MIN6 (e.g. Fig. 2B and 2E). A previous study has shown that Yap1 expression was lost following endocrine specification in developing mouse embryos and Yap1 was hardly detected in mouse adult β-cells through immunohistochemistry (George et al., 2012). The level of Yap1 protein in mouse adult islets was relatively lower compared to other cell types in the pancreas, which may have led to an underestimate of Yap1 expression in β-cells. Also, it is possible that gene expression profiles differ between primary β-cells and insulinoma cell lines.

While FFA reduces β-cell viability, we found that Yap1 is activated in response to FFA treatment as a way for the cells to minimize the negative impact of FFA on cell survival. We demonstrated a positive role of Yap1 in maintaining β-cell viability on the basis of following three lines of evidence. First, loss-of-function analysis of Yap1 indicated that Yap1 does not significantly promote proliferation, but is critical for suppressing apoptosis of INS-1 832/13 cells (Fig. 2H and 2I). Absence of Yap1 synergistically increased apoptosis in cells treated with FFA (Fig. 2K). This protective role of Yap1 was further supported by the gain-of-function analysis of Yap1. Increasing the level of Yap1 suppressed apoptosis both under normal and FFA-treated conditions in INS-1 832/13 cells (Fig. 2L).

Second, upon FFA treatment, Yap1 became dephosphorylated and translocated into the nucleus (Figs. 2B, 2C, 2E, 2F and S2A). In many cell types, the activity of Yap1 is regulated by a tumor-suppressive pathway called the Hippo (Hpo) signaling pathway. In the Hpo pathway, the activity of Yap1 gets suppressed when the large tumor suppressor 1/2 (Lats1/2) phosphorylate Yap1 for its cytoplasmic retention and proteasomal degradation (Zhao et al., 2010). In this study, we tested whether Lats1 regulates Yap1 activity under the FFA-mediated stress condition in β-cells. To address this question, we used the phosphorylation level of Lats1 (at the conserved Ser909) as a read-out of Lats1 activity. We showed that Lats1 was dephosphorylated after palmitate treatment, and this palmitate-induced dephosphorylation was partially blocked by the disruption of F-actin (Fig. S2C). This Lats1 activity pattern was consistent with that of Yap1 phosphorylation, supporting a model that Lats1 kinase is responsible for the phosphorylation of Yap1. In the canonical Hpo pathway, the mammalian STE20-like protein kinases 1/2 (Mst1/2) phosphorylate Lats1/2 for regulating their kinase activity (Zhao et al., 2010). To test whether Mst1/2 is involved in regulating Lats1/2 activity in β-cells, we monitored the phosphorylation levels of Mst1 (at Thr183) and Mst2 (at Thr180). Surprisingly, the phosphorylation level of Mst1/2 increased upon palmitate treatment and co-treatment with CytoD (Fig. S2D). Activation of Mst1/2 under various stress conditions, including palmitate, in pancreatic islets and a β-cell line has been reported (Ardestani et al., 2014). This suggests the reduction of the phosphorylation level of Lats1 upon palmitate treatment is not due to the inactivation of Mst1/2. Taken together, although calling for more investigation, this Yap1’s response may be regulated by its upstream kinase, Lats1/2, in an Mst1/2-independent manner.

Third, expression of a well-established Yap1 downstream target gene, CTGF, increased upon FFA treatment and Yap1 is required for this up-regulation in INS-1 832/13 cells (Fig. 4C–F). Gain-of-function analysis of CTGF exhibited its protective role against FFA-induced apoptosis (Fig. 4G and 4H). Therefore, we propose a model that Yap1 activation is a critical step of cellular response to FFA signaling and activated Yap1 suppresses apoptosis through its downstream target genes such as CTGF. The CTGF gene was identified as a direct YAP/TEAD target required for cell growth (Zhao et al., 2008). In the mouse embryonic pancreas, CTGF functions in both endothelial cells and β-cells, and is both required and sufficient to promote β-cell proliferation (Guney et al., 2011). However, in adult pancreas, CTGF expression is lost in β-cells and is only confined to ductal cells and microvessels with in the islets (Crawford et al., 2009). Importantly, we found that CTGF expression can be increased in FFA-treated islets derived from adult mice (data not shown). Our finding of exogenous CTGF promoting β-cell viability under FFA treatment without the induction of proliferation makes CTGF a good candidate for potential diabetic treatment.

We also showed that the palmitate-induced apoptosis is mediated by F-actin dynamics. Our results suggest that F-actin dynamics plays a key role in activating a protective mechanism against FFA-induced stress in β-cells. When F-actin inhibitors were added along with palmitate, the induction of apoptosis was partially blocked (Fig. 1C). Yap1 activation provides the molecular basis of this protective mechanism (Figs. 3 and S2). It is intriguing that the disruption of F-actin polymerization was no longer effective in suppressing FFA-induced apoptosis if the F-actin inhibitor, CytoD, was added at a later stage, a day after FFA treatment. Understanding the mechanism of how actin cytoskeleton regulates the FFA-induced apoptosis needs more investigation.

FFAs can act through GPR40 to influence insulin secretion by β-cells (Steneberg et al., 2005). However, whether GPR40 also mediates FFA-induced apoptosis in β-cells remains unclear. In mouse NIT-1 β-cells, palmitate-induced apoptosis was not mediated by GPR40 (Zhang et al., 2007), while another group found that GPR40 was critical for palmitate-induced ER stress and apoptosis in mouse MIN6 β-cells (Wu et al., 2012). To explore if palmitate can also act on GPR40 to induce apoptosis in rat INS-1 cells, we used a selective GPR40 antagonist, GW1100, to inhibit GRP40 function. The result showed that the loss of GPR40 function did not prevent palmitate-induced apoptosis; instead, GW1100 treatment at higher concentrations moderately enhanced palmitate-induced apoptosis (data not shown).

Although FFA signaling might directly lead to Yap1 activation, we cannot exclude the possibility that the effect of FFA on Yap1 activation is indirect. Other signaling pathways, such as JNK and p38 MAPK pathways, might be deployed upon FFA treatment to modulate F-actin or other mechanisms to cause Yap1 activation in β-cells (Natalicchio et al., 2013). Supporting the latter model, Yap1 activation in β-cells appears to occur after apoptosis has been initiated. *In vivo*, apoptotic cells are known to be able to send out proliferative and survival signals through paracrine as well as autocrine signaling (e.g. Fan and Bergmann, 2008; Sun and Irvine, 2011). In fact, CTGF is a secretory protein and could be possibly involved in such a mechanism *in vivo*.

## MATERIALS AND METHODS

### Cell culture

Rat INS-1 832/13 and mouse MIN6 β-cells were kindly provided from Dr. Douglas Cavener’s laboratory, Pennsylvania State University (Wang et al., 2013). INS-1 832/13 cells were cultured in RPMI 1640 medium (Lonza) with 2.1 mmol/L L-glutamine and 11.1 mmol/L D-glucose (Lonza) supplemented with 10% FBS (Gemini), 10 mmol/L HEPES (Lonza), 1 mmol/L NaPyruvate (Thermo Scientific), and 55 µmol/L β-Mercaptothanol (ME) (Gibco in Invitrogen). MIN6 cells were cultured in DMEM medium (Invitrogen) with 25 mmol/L D-glucose supplemented with 15% FBS, 10 mmol/L HEPES, and 55 µmol/L β-ME. The cells were cultured in 37°C with 5% CO_2_ and medium was changed every two days. 1:3 dilution was used to pass the cells when they grow to at least 80% confluence.

### Chemical treatment

Palmitate (Sigma) was dissolved in ethanol and was stored as 50 mmol/L solution at −20°C. To assess the effects of FFAs, palmitate was pre-added to 50 µL culture medium, heated to 70°C for complete dissolution, and then added the whole 50 µL mixture to the cells. Cytochalasin D (CytoD) (Sigma) was dissolved in DMSO (dimethyl sulfoxide) and stored as 2 mmol/L solution at −20°C. Latrunculin B (LatB) (Millipore) was dissolved in DMSO and stored at 4 mg/mL solution at −20°C. A final concentration of 1 µg/mL of recombinant human CTGF protein (Gemini) was used to treat β-cells under cultured conditions.

### Annexin V staining and flow cytometry

For apoptosis analysis, cells were trypsinized from 24 well plates and stained using Alex Fluor® 488 Annexin V kit (Invitrogen) following manufacturer’s protocol. Flow cytometry data were collected by FC 500 (Beckman Coulter, Inc.).

### Overexpression and knockdown of Yap1

Both overexpression and knockdown of Yap1 was done by lentiviral infection. For knockdown of Yap1, pLKO.1 vector expressing shRNA of Yap1 (a gift from Dr. Kun-Liang Guan) was transfected together with pMD2.G and psPAX2 in HEK 293T cells to produce lentiviral particles. For overexpression of Yap1, pCDH-CMV-MCS-EF1-RFP vector expressing human Yap1 was transfected together with VSVG and Pax2 plasmids in HEK 293T cells to produce lentiviral particles (gifts from Dr. Yingwei Mao). Lentiviral particles were infected into INS-1 832/13 cells with 8 µg/mL polybrene.

### RNA extraction and quantitative reverse transcription polymerase chain reaction

After chemical treatments of INS-1 832/13 cells, total mRNA was extracted with RNeasy Mini Kit (Qiagen). Extracted RNA was reverse transcribed to cDNA with qScipt^TM^ cDNA SuperMix (Quanta) by following manufacturer’s instructions. Quantitative RT-PCR was performed using PerfeCTa^®^ SYBR^®^ Green FastMix, Rox^TM^ (Quanta) and the StepOnePlus^TM^ Real-Time PCR system (Applied Biosystems). Gene expression levels were normalized to Tubulin of the same sample. Sequences of rat primers used in the experiment are listed below, Yap1: forward 5′ TCGTTTTGCCATGAACCAGA, reverse 5′ GGCTGCTTCACTGGAGCACT; Bax: forward 5′ GGATACAGACTCCCCCCGAG, reverse 5′ AACATGTCAGCTGCCACACG; Pml: forward 5′ GCACCATCCCTGAAAAAGAGAC, reverse 5′ TGGCCACATGGTTGTTGTTG; Ctgf: forward 5′ TCTTCGGTGGGTCCGTGTAC, reverse 5′ TGCAACTGCTTTGGAAGGACT; Cyr61: forward 5′ ATTGGCAAAGGCAGCTCACT, reverse 5′ AGGTTCCGTGCCAAAGACAG; Axl: forward 5′ CTGTGATGGAGGGCCAGCT, reverse 5′ CAGCGACCTTGAGGATGGAG; Tubulin: forward 5′ GAAGTTCGCACTGGCACCTAC, reverse 5′ GATGAGCTGCTCTGGGTGGA.

### Western blot analysis

Western blot was done by running 10% SDS-PAGE, following standard blotting protocol. Primary antibodies used in this study include: rabbit anti-cleaved Caspase 3 (1:1000, Cell Signaling), rabbit anti-YAP (1:1000, Santa Cruz), rabbit anti-Phospho-YAP (1:500, Cell Signaling), rabbit anti-Lats1 (1:500, Cell Signaling), rabbit anti-Phospho-Lats1 (1:500, Cell Signaling), rabbit anti-Phospho-Mst1/2 (1:500, Cell Signaling), and mouse anti-α-Tubulin (1:2000, Sigma) antibodies. Secondary antibodies are donkey anti-rabbit or mouse IgG antibodies (Amersham).

### Immunocytochemistry, flow cytometry and microscopy

For immunocytochemistry, cells were grown on coverslips in 24-well plates until they became confluent. After chemical treatments, cells were fixed with 4% PFA, incubated with a primary antibody at room temperature for 1 h, and then incubated with a secondary antibody in foil wrap for 1 h at room temperature. For flow cytometry assays, cells were grown directly on the 24-well plates. After chemical treatment, cells were trypsinized and harvested before staining. Antibodies and chemicals used in this study include: anti-YAP (1:200, Santa Cruz), anti-Phospho-Histone 3 (PH3) (1:100, Cell signaling), Alexa Fluor 568, Alexa Fluor 594 (1:200, Invitrogen), Draq 5 (1:400, Cell Signaling), DAPI (4’,6-diamidino-2-phenylindole) (1:200, Invitrogen), and Phallotoxin (1:200, Invitrogen). Images were collected by Olympus Fluoview 300 and Olympus Fluoview 1000 confocal laser scanning microscopes. Flow cytometry data were collected with an FC 500 cytometer (Beckman Coulter, Inc.).

### Statistical analysis

Statistical analysis was performed using Student’s *t*-test on Microsoft Excel.

## Electronic supplementary material

Below is the link to the electronic supplementary material.

Supplementary material 1 (PDF 616 kb)
