# Supplementary material for: Yap1 plays a protective role in suppressing free fatty acid-induced apoptosis and promoting beta-cell survival
Source: Protein Cell. 2016 Mar 22;7(5):362–72. doi: 10.1007/s13238-016-0258-5 (PMC4853318; doi:10.1007/s13238-016-0258-5)
Supplement: Supplementary file 1 — Supplementary material 1 (PDF 616 kb) [file 13238_2016_258_MOESM1_ESM.pdf]

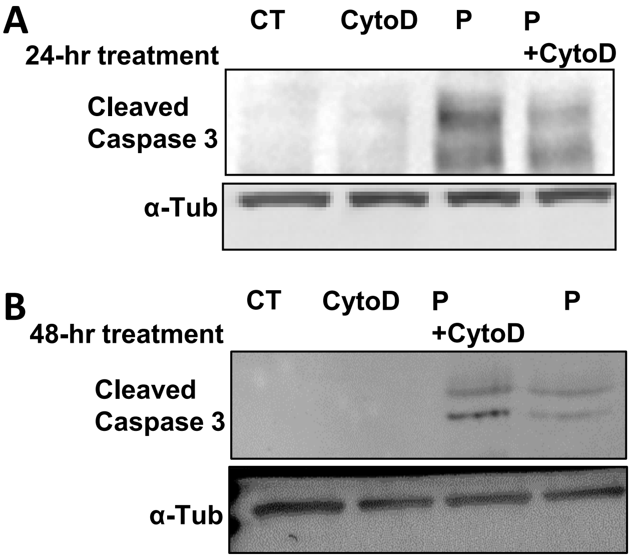

Supplementary Figure 1. F-actin modulation is critical for palmitate-induced β-cell apoptosis. (**A**) Western blot analysis of cleaved (activated) Caspase 3 shows that CytoD inhibits palmitate (P)-induced apoptosis under 24-hour treatment. (**B**) Western blot targeting cleaved Caspase 3 shows that CytoD amplifies palmitate-induced apoptosis signal under 48-hour treatment. In both (A) and (B), control experiments used solvents (ethanol and DMSO). α-Tubulin (Tub) level was used for loading control. CT: control; CytoD: Cytochalasin D; P: Palmitate.

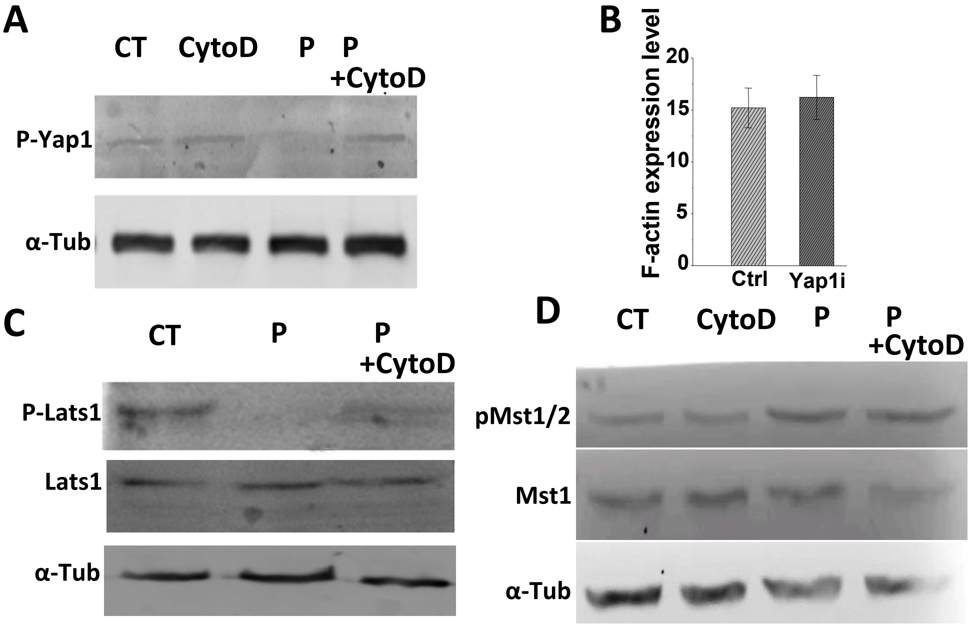

Supplementary Figure 2. Yap1 activity is regulated by F-actin under palmitate treatment and Lats1, but unlikely Mst1/2 kinases. (**A**) Western blot analysis with a phospho-specific antibody against Yap1 (at Ser127) shows that Yap1 was dephosphorylated under 24-hour palmitate treatment. INS-1 832/13 cells were treated with palmitate alone or palmitate together with CytoD for 24 hours. Dephosphorylation of Yap1 was inhibited by CytoD. (**B**) Loss of Yap1 had no obvious effect on F-actin level. F-actin was quantified by flow cytometry after phallotoxin staining. Ctrl: a negative control. Yap1i: Yap1 shRNA. Data shows mean ± S.D. of four independent experiments. (**C**) Palmitate treatment inactivates Lats1 activity via F-actin. Western blot analysis with a phospho-specific antibody against active Lats1 kinase (at Ser909) shows that Lats1 was dephosphorylated upon palmitate treatment, and this dephosphorylation was partly inhibited by CytoD. (**D**) Western blot analysis with a phospho-specific antibody against active Mst1/2 (at Thr183 for Mst1 and at Thr180 for Mst2) shows that the levels of active Mst1/2 were increased by palmitate or palmitate+CytoD treatment. In (A), (C) and (D), solvents (ethanol and DMSO) were used in the control experiments. α-Tubulin (Tub) level was used for loading control. CT: control; CytoD: Cytochalasin D; P: Palmitate.
